# Differential Cytokine Gene Expression in Granulomas from Lungs and Lymph Nodes of Cattle Experimentally Infected with Aerosolized *Mycobacterium bovis*

**DOI:** 10.1371/journal.pone.0167471

**Published:** 2016-11-30

**Authors:** Mitchell V. Palmer, Tyler C. Thacker, W. Ray Waters

**Affiliations:** Infectious Bacterial Diseases of Livestock Unit, National Animal Disease Center, Agricultural Research Service, United States Department of Agriculture, Ames, Iowa, United States of America; Fundació Institut d’Investigació en Ciències de la Salut Germans Trias i Pujol, Universitat Autònoma de Barcelona, SPAIN

## Abstract

The hallmark lesion of tuberculosis in humans and animals is the granuloma. The granuloma represents a distinct host cellular immune response composed of epithelioid macrophages, lymphocytes, and multinucleated giant cells, often surrounding a caseous necrotic core. Within the granuloma, host-pathogen interactions determine disease outcome. Factors within the granulomas such as cytokines and chemokines drive cell recruitment, activity, function and ultimately the success or failure of the host’s ability to control infection. Hence, an understanding of the granuloma-level cytokine response is necessary to understand tuberculosis pathogenesis. *In-situ* cytokine expression patterns were measured using a novel *in situ* hybridization assay, known as RNAScope^®^ in granulomas of the lungs, tracheobronchial lymph nodes and caudal mediastinal lymph nodes of cattle experimentally infected with *Mycobacterium bovis* via aerosol exposure. In spite of microscopic morphological similarities, significant differences were seen between late stage granulomas of the lung compared to those of the tracheobronchial lymph nodes for IL-17A, IFN-γ, TGF-β, IL10 and IL-22 but not for TNF-α. Additionally, significant differences were noted between granulomas from two different thoracic lymph nodes that both receive afferent lymphatics from the lungs (i.e., tracheobronchial and caudal mediastinal lymph nodes) for TNF-α, IL-17A, IFN-γ, TGF-β and IL-10 but not for IL-22. These findings show that granuloma morphology alone is not a reliable indicator of granuloma function as granulomas of similar morphologies can have disparate cytokine expression patterns. Moreover, anatomically distinct lymph nodes (tracheobronchial vs caudal mediastinal) differ in cytokine expression patterns even when both receive afferent lymphatics from a lung containing tuberculoid granulomas. These findings show that selection of tissue and anatomic location are critical factors in assessing host immune response to *M*. *bovis* and should be considered carefully.

## Introduction

Bacteria of the genus *Mycobacterium* are Gram-positive, acid-fast bacilli that include several major human and animal pathogens [1]. Tuberculosis in humans is caused by *Mycobacterium tuberculosis*; however, indistinguishable disease can be caused by the zoonotic agent *Mycobacterium bovis* [2]. *Mycobacterium bovis* is a member of the *Mycobacterium tuberculosis* complex, which also includes *M*. *tuberculosis*, *M*. *bovis*, *M*. *africanum*, *M*. *microti*, *M*. *caprae*, *M*. *canetti*, *M*. *pinnipedii* and *M*. *mungi* [3]. The range of susceptible hosts to *M*. *bovis* is extremely broad and includes humans, domestic and wild ruminants, swine and carnivores. Generally speaking, the hallmark lesion of tuberculosis, regardless of host or tissue, is the *granuloma*. A granuloma that results from infection with mycobacteria belonging to the *M*. *tuberculosis* complex may also be referred to as a *tubercle* or *tuberculoid granuloma* [4].

The pathogenesis of bovine tuberculosis parallels many aspects of human tuberculosis and has been a useful model of human tuberculosis [5, 6]. The pathogenesis of primary human or bovine tuberculosis involves lungs and regional lymph nodes in a coordinated exchange of cells under the influence of cytokines, chemokines and other mediators. Following inhalation, bacilli are deposited in the terminal bronchioles and alveoli where they are phagocytosed by resident macrophages, inducing the production of cytokines, chemokines and enzymes. Both pro (e.g., IFN-γ, TNF-α, IL-2) and anti-inflammatory (e.g., IL-10, TGF-β) cytokines are produced by resident macrophages and activate innate immune cells such as neutrophils, monocytes, macrophages and dendritic cells [7]. The adaptive immune response is initiated as dendritic cells containing bacilli migrate from the lung to regional lymph nodes, activating naïve T-cells through antigen presentation and cytokine production. Following their activation and expansion, T-cells home to the site of infection in the lungs, and with epithelioid macrophages and multinucleated giant cells form a granuloma [7]. The association of lung and regional lymph nodes in tuberculosis pathogenesis has long been noted, as illustrated by identification of the primary complex, or Ghon complex (named for Anton Ghon (1866–1936)), which is defined as the presence of both a primary pulmonary lesion and a caseous lymph node lesion [8].

The tuberculoid granuloma is dynamic in nature. Individual granulomas develop in an independent manner with diverse trajectories, influenced by local immunity and mycobacterial growth; thereby disease is ultimately controlled at the granuloma level [9]. We have shown in cattle, the presence of morphologically diverse granulomas in the lungs and lymph nodes, categorizing them into four stages based on microscopic determination of cellular composition, size, necrosis, fibrosis and mineralization [10–12]. Briefly, these stages are defined as follows: initial (stage I), solid (stage II), necrotic (stage III) and necrotic and mineralized (stage IV).

Within the granuloma, key interactions between host and pathogen determine disease confinement or dissemination, bacterial replication, killing or latency [11, 13]; therefore, it is imperative to understand granuloma-level immune responses. Studies using cattle have described granuloma-level immune responses by demonstrating the presence of cytokines, chemokines and enzymes using immunohistochemistry (IHC) or laser capture microdissection (LCM) of granulomas combined with quantitative real time PCR (qPCR) [10, 14–17]. Other studies have even focused on granulomas of different morphologic stages [14, 15, 17–20].

The heterogeneity of tuberculoid granulomas and the involvement of multiple organs (i.e., lung and lymph nodes) suggest that host responses within different granulomas are likely varied, not only within an organ but between organs as well. Indeed, in bovine tuberculosis, pulmonary granulomas have been shown to produce more IFN-γ and IL-17A than those found in lymph nodes of the same animal [21]. Studies of bovine tuberculosis to date have not focused on granulomas of different stages in different organs (e.g. lung and lymph nodes).

The objective of this study was to quantify expression of various cytokines of known or implied relevance to bovine tuberculosis in granulomas of various stages in the lungs and lymph nodes (tracheobronchial and caudal mediastinal) of cattle experimentally infected with aerosolized *M*. *bovis*. Alternatively expressed from a histopathological perspective, the objective was to determine if granulomas of the same morphologic stage (i.e., I to IV) represent similar or distinct cytokine environments when present in the lungs compared to the lymph nodes.

## Results

**Fig 1:**
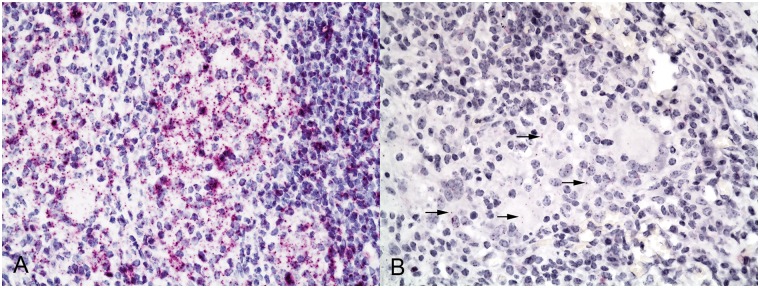
Expression of A) TGF-β and B) IL-10 mRNA in lung granulomas of calves experimentally infected with *Mycobacterium bovis* by aerosol. Note signal (red dots) representing mRNA transcripts within cells of various types. The amount of signal varied widely between cytokines (arrows). RNAScope^®^ ISH, 400X.

All experimentally infected calves were considered “reactors” based on USDA definitions for interpretation of the comparative cervical test (CCT) [22]. Likewise, all experimentally infected calves developed gross and microscopic lesions in at least 1 lung lobe. One calf had no gross lesions in pulmonary associated lymph nodes, but did have microscopic lesions. All other calves had gross lesions in both tracheobronchial and caudal mediastinal lymph nodes. For all cytokines or chemokines examined, as well as positive control targets, labeling of mRNA transcripts was represented as dots of red chromogenic substrate (i.e. Fast Red). The number of dots within a single cell varied from several dots to numerous dots (Fig 1), sometimes coalescing to form an amorphous cluster of intense labeling. The size of a single dot varied from approximately 0.2 to 1.5 μm. Within granulomas, cells morphologically consistent with lymphocytes, macrophages and multinucleated giant cells contained some degree of labeling for all chemokines and cytokines examined.

A total of 37 lung, 88 tracheobronchial lymph node and 100 caudal mediastinal lymph node granulomas were analyzed. Late stage granulomas represented 87% (32/37), 50% (44/88) and 50% (50/100) of lung, tracheobronchial lymph node and caudal mediastinal lymph node granulomas, respectively. As the preponderance (87%) of granulomas in the lungs were categorized as late stage IV, comparisons between lung and tracheobronchial lymph node were limited to late stage granulomas.

### Lung vs tracheobronchial lymph node

**Fig 2:**
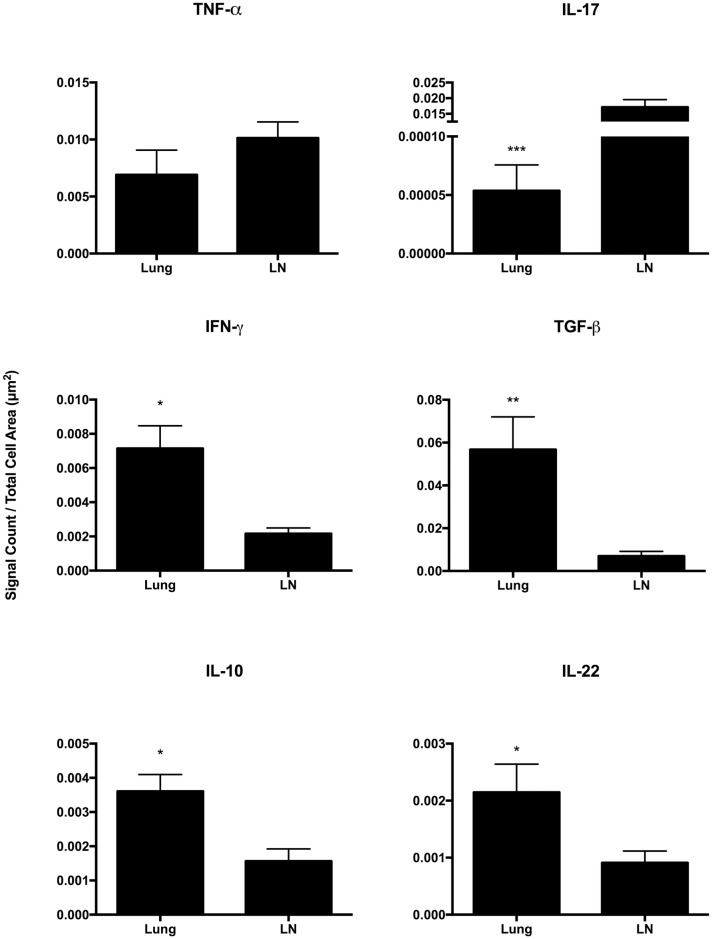
Cytokine gene expression in late stage granulomas of the lung vs tracheobronchial lymph node (LN) of calves experimentally infected with *Mycobacterium bovis*. Gene expression as measured by quantification of mRNA transcripts identified through RNAScope^®^ ISH. Values represent the mean ± SEM mRNA transcripts/total cell area (μm^2^) of granuloma. Significantly different * (*p*< 0.05), ** (*p*< 0.001), *** (*p*< 0.0001).

Differential cytokine expression was seen in granulomas of the lungs compared to those of the tracheobronchial lymph nodes for all cytokines examined with the exception of TNF-α (Fig 2). Gene expression in lung granulomas was greater than that in tracheobronchial lymph node granulomas for IFN-γ, TGF-β, IL-10 and IL-22. In contrast, expression of IL-17A was greater in granulomas of the tracheobronchial lymph nodes compared to those of the lung.

### Tracheobronchial lymph node vs caudal mediastinal lymph node

**Fig 3:**
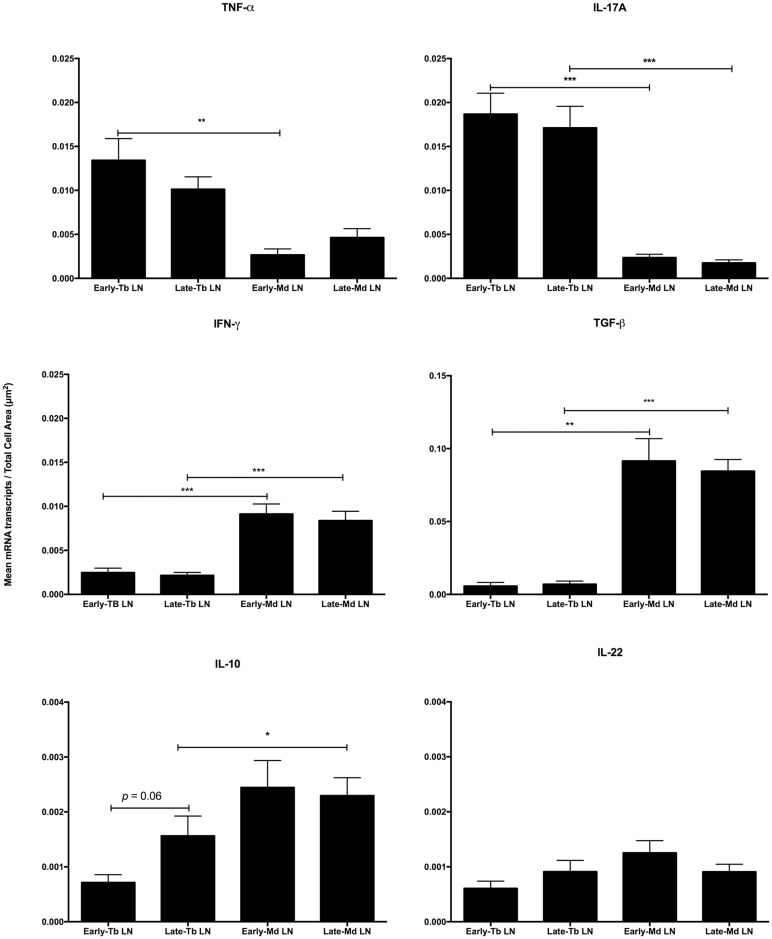
Cytokine gene expression in early (stage I) vs late (stages III and IV) granulomas of the tracheobronchial lymph node (Tb LN) vs caudal mediastinal lymph nodes (Md LN) of calves experimentally infected with *Mycobacterium bovis*. Gene expression as measured by quantification of mRNA transcripts identified through RNAScope^®^ ISH. Values represent the mean ± SEM mRNA transcripts/total cell area (μm^2^) of granuloma. Significantly different * (*p*< 0.05), ** (*p*< 0.001), *** (*p*< 0.0001).

To examine cytokine gene expression in two different thoracic lymph nodes, commonly involved in bovine tuberculosis, and in some studies pooled for analyses, we compared cytokine gene expression in granulomas of the tracheobronchial lymph node and caudal mediastinal lymph node. Differential cytokine gene expression was seen between the two nodes for TNF-α, IL-17A, IFN-γ, IL-10 and TGF-β (Fig 3). Expression of TNF-α and IL-17A were greater in tracheobronchial lymph nodes while IFN-γ, TGF-β, and IL-10 were greater in caudal mediastinal lymph nodes. Within lymph node locations, significant differences between early and late stage granulomas were not seen for any of the cytokines examined, with one exception that approached statistical significance; expression of IL-10 was higher (*p* = 0.06) in late stage granulomas compared to early stage granulomas in the tracheobronchial lymph nodes (Fig 3).

### Granuloma vs non-granuloma containing tissue

**Fig 4:**
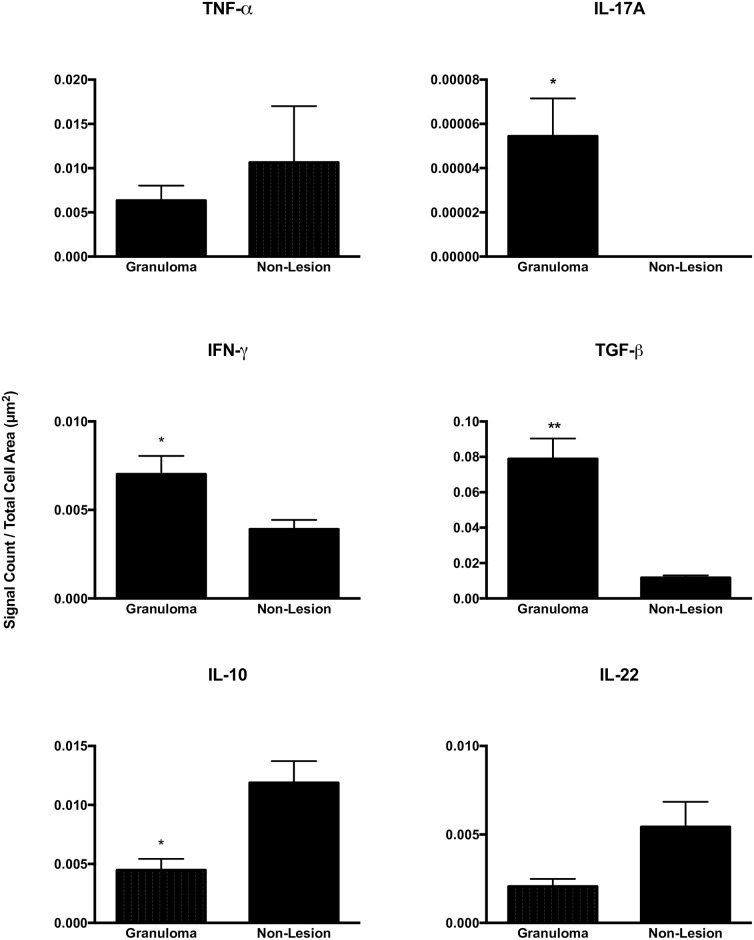
Cytokine gene expression within granulomas of the lung vs non-granuloma (non-lesion) regions on the same slide from calves experimentally infected with *Mycobacterium bovis*. Gene expression as measured by quantification of mRNA transcripts identified through RNAScope^®^ ISH. Values represent the mean ± SEM mRNA transcripts/total cell area (μm^2^) of granuloma. Significantly different * (*p*< 0.05), ** (*p*< 0.001), *** (*p*< 0.0001).

**Fig 5:**
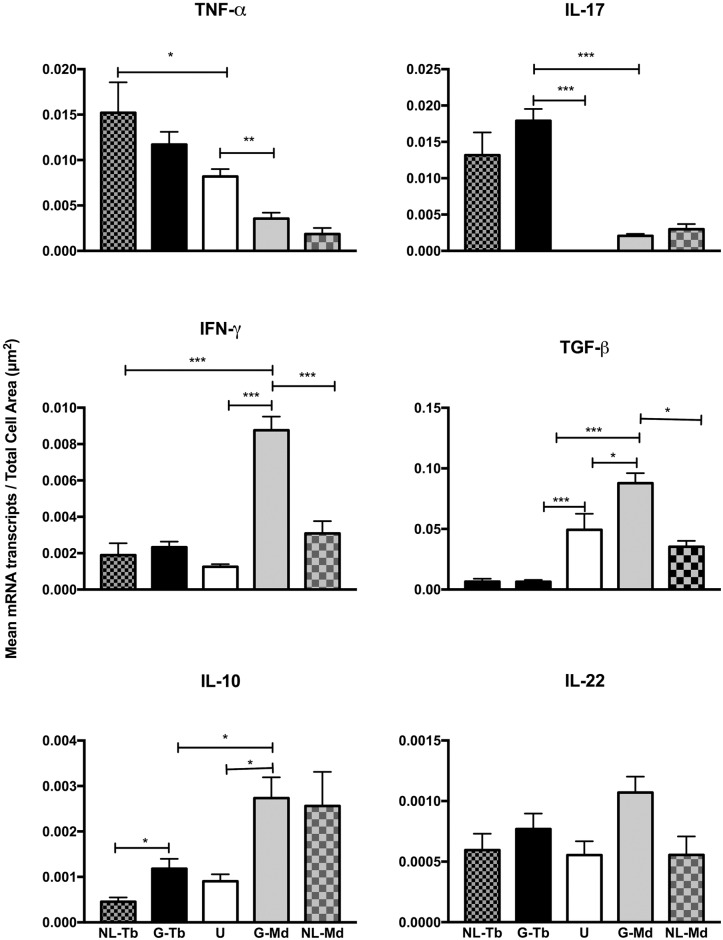
Cytokine gene expression within granulomas of the tracheobronchial (G-Tb) and caudal mediastinal (G-Md) lymph nodes vs non-granuloma (non-lesion) regions (NL-Tb, NL-Md) on the same slide from calves experimentally infected with *Mycobacterium bovis*. **Uninfected (U) caudal mediastinal lymph nodes from age matched calves are shown for comparison.** Gene expression as measured by quantification of mRNA transcripts identified through RNAScope^®^ ISH. Values represent the mean ± SEM mRNA transcripts/total cell area (μm^2^) of granuloma. Significantly different * (*p*< 0.05), ** (*p*< 0.001), *** (*p*< 0.0001).

Lungs and lymph nodes were also analyzed to compare, on the same slide, cytokine gene expression within granulomas compared to non-granuloma areas where tissue appeared histologically normal. Within the lung, gene expression was significantly greater within granulomas compared to non-granuloma areas for IL-17A, IFN-γ and TGF-β (Fig 4). In contrast, expression was less within lung granulomas compared to non-granuloma areas for IL-10. Among tracheobronchial lymph nodes only IL-10 differed in levels of expression between granulomas and non-granuloma areas, with expression being greater within granulomas than in non-granuloma areas (Fig 5). Among caudal mediastinal lymph nodes, expression of IFN-γ and TGF-β were significantly higher within granulomas than in non-granuloma areas (Fig 5).

### Lymph node granulomas vs uninfected lymph node

Comparisons were also made between lymph node granulomas and uninfected caudal mediastinal lymph nodes from age-matched calves. For TNF-α, expression was greater in tracheobronchial lymph node granulomas, but less in caudal mediastinal lymph granulomas compared to uninfected caudal mediastinal lymph nodes (Fig 5). For IL-17A, expression in granulomas of only tracheobronchial lymph nodes was greater than that seen in uninfected caudal mediastinal lymph nodes. For IFN-γ and IL-10, expression was greater only in the caudal mediastinal lymph node granulomas compared to uninfected caudal mediastinal lymph node. For TGF-β, expression was greater in caudal mediastinal lymph node granulomas, but less in tracheobronchial lymph node granulomas compared to uninfected caudal mediastinal lymph nodes. No differences in IL-22 expression were seen between lymph node granulomas and uninfected lymph node.

## Discussion

The present study demonstrates differential cytokine gene expression in granulomas from the lungs compared to granulomas from the tracheobronchial lymph nodes in spite of their morphological similarities. Moreover, cytokine gene expression differed between two lymph nodes (tracheobronchial and caudal mediastinal); both found in the thoracic cavity and commonly associated with lesions of bovine tuberculosis. These findings highlight the importance of anatomic location when evaluating local host immune responses to *M*. *bovis*.

Differential cytokine expression may result from the independent nature of the granuloma microenvironment. Using the macaque model of human tuberculosis, it is shown that even when examined at a single time point in disease progression, granulomas are independent and differ in terms of cytokine expression patterns, cell numbers, cell types and bacterial burden [9, 23]. Additionally, it has been shown in *M*. *tuberculosis*-infected humans that mycobacterial antigen expression differs in granulomas of the lungs compared to that of the lymph nodes [20] potentially resulting in variable pathogen related stimuli inciting different host responses based on the organ affected. Further work, focused on individual granulomas will be required to determine if bovine tuberculoid granulomas demonstrate the same level of heterogeneity as those seen in the macaque model. Nonetheless, it has previously been observed that bovine tuberculoid granulomas vary in morphology at both the gross and microscopic levels. These morphologic differences have been used to score gross lesion severity and to categorize granulomas into 4 different microscopic stages (I-IV). Characteristics such as necrosis, mineralization and fibrosis are properties of stages III and IV and are considered attributes of disease progression and increased disease severity [10, 11, 15, 16, 24, 25]. Although useful in categorizing disease progression or severity, the present study shows that conclusions on granuloma function based solely on morphological characteristics must be made with caution.

Previous studies on cytokine gene or protein expression in bovine tuberculoid granulomas have reported results from individual pulmonary lymph nodes (i.e. tracheobronchial or mediastinal) [26], other studies have not identified which thoracic lymph nodes were examined or have pooled findings from both tracheobronchial and mediastinal lymph nodes [14, 16, 19, 21]. Disparate cytokine expression patterns between tracheobronchial and caudal mediastinal lymph nodes, as seen in the present study may be partially due to the afferent lymphatics serving these closely associated lymph nodes. In cattle, the tracheobronchial lymph node lies near the tracheal bifurcation and receives afferent lymphatics from the lungs, esophagus and heart. More caudally, the caudal mediastinal lymph node lies between right and left lung lobes, alongside the esophagus and aorta. It is the largest of the mediastinal nodes and in addition to lungs, esophagus and heart, receives afferent lymphatics from the trachea, thymus, pleura, diaphragm, liver and spleen [27]. The distinct environments from which afferent lymphatics originate likely influences both cells and signals reaching these two thoracic lymph nodes, thereby altering cytokine expression patterns. Given the disparate cytokine expression patterns and differences in regional lymphatic drainage, pooling tracheobronchial and mediastinal lymph nodes in order to evaluate “pulmonary lymph node” responses in bovine tuberculosis may yield misleading results.

The cytokines examined in the present study are of known or implied relevance to bovine tuberculosis pathogenesis. The causes or effects of increased or decreased expression in lesions of one organ over another cannot be inferred from the present data. However, IFN-γ has been demonstrated in bovine tuberculoid lymph node granulomas using qPCR and IHC [14, 16, 26, 28, 29]. IFN-γ is a principal mediator of macrophage activation and considered pivotal in a protective Th1 response to mycobacteria. TNF-α is key in controlling the organization of myeloid and lymphoid cells into granulomas capable of containing mycobacterial proliferation [30]. Low levels of TNF-α are associated with fatal disease progression [31], while excessive TNF-α induces a hyper-inflammatory state, which promotes tissue damage and destruction [30]. IL-10 is an important anti-inflammatory cytokine controlling innate and adaptive immune responses to tuberculous mycobacteria [7]. The principal function of IL-10 is to deactivate macrophages, resulting in diminished Th1 cytokine production, as well as decreased production of reactive nitrogen and oxygen species [32]. IL-17A is produced by T-cells, a subset of which are known as Th17 cells, and using qPCR, IL-17A has been detected in bovine tuberculoid lymph node and pulmonary granulomas [14, 21] but a comparison of lung and lymph node expression has not been done. IL-22 responses in cattle vaccinated with *M*. *bovis* BCG have been correlated with vaccine-induced protection [33]. In experimentally infected cattle, using LCM and qPCR, IL-22 mRNA expression showed a decreasing trend from stage I through stage IV using a pool of tracheobronchial, bronchial and both cranial and caudal mediastinal lymph nodes for examination [14]. TGF-β may play an anti-inflammatory role, downregulating a Th1 immune response. In bovine tuberculoid lymph node granulomas TGF-β has been demonstrated by IHC and ISH in epithelioid macrophages and Langhans multinucleated giant cells; however, quantification of expression has revealed inconsistent results. One study found greater TGF-β by IHC in late stage bovine granulomas compared to early stage granulomas [10]. Two separate studies found lower expression of TGF-β mRNA in stage IV granulomas of cattle compared to stage I granulomas [19] with increased expression in stage II granulomas [14]. In macaques, TGF-β may be a chief cytokine in driving tissue repair in active tuberculosis [34]. It should be remembered that TGF-β is produced in an inactive form that must be proteolytically cleaved to become functional [35]. Therefore, demonstration of TGF-β mRNA may not be an accurate reflection of TGF-β biological activity.

The present study demonstrates that morphology alone is not a reliable indicator of granuloma function as late stage granulomas in the lung displayed a very different cytokine expression pattern than late stage granulomas in the tracheobronchial lymph nodes. Moreover, in analyzing immune responses of lungs and associated lymph nodes, the choice of lymph node is important. With such different cytokine expression patterns as those measured in the present study it may not be appropriate to pool tracheobronchial and mediastinal lymph nodes for analysis.

## Materials and Methods

### *Mycobacterium bovis* aerosol challenge

Inoculum preparation and details of experimental aerosol infection of calves with virulent *M*. *bovis* have been described in detail previously [18] [36]. Briefly, castrated male Holstein calves (3 months of age) were obtained from a tuberculosis-free herd near State Center, Iowa. After 2 weeks of outside housing calves were moved into biosafety level 3 (BSL-3) housing for acclimation. After 8 weeks of acclimation calves were infected with virulent *M*. *bovis* by nebulization of inoculum into a mask (Equine AeroMask^®^, Trudell Medical International, London, ON, Canada) covering the nostrils and mouth. The aerosol mask is well tolerated, requiring only manual restraint using traditional livestock head gates. Calves received either 10^4^ colony-forming units (cfu) of *M*. *bovis* 95–1315 (n = 8) or 10^4^ cfu *M*. *bovis* 10–7428 (n = 8) by aerosol as described [37]. Both are field strains and have been shown to be equally virulent in the calf aerosol model [36]. Archived samples from 5 age-matched, non-infected calves from the same source herd were used as a source of uninfected caudal mediastinal lymph nodes. Ten days prior to euthanasia, tuberculin skin testing was done on *M*. *bovis* infected calves according to guidelines described in USDA APHIS circular 91-45-01 [22] for the comparative cervical test (CCT). Briefly, two separate sites on the lateral neck were clipped of hair, skin thickness measured with calipers and 0.1 ml (100 μg) of *M*. *bovis* purified protein derivative (PPD) or 0.1 ml (40 μg) *M*. *avium* PPD was intradermally injected into one site each. Skin thickness was measure at 72 hours after injection to quantify delayed type hypersensitivity reactions to PPDs. All experimental procedures involving animals were conducted in accordance with recommendations in the Care and Use of Laboratory Animals of the National Institutes of Health. The USDA-National Animal Disease Center’s Institutional Biosafety and Animal Care and Use Committee approved the experiment, protocol ARS-2015-451. Throughout the study, animals were monitored twice daily by animal care staff. In outside housing, calves were fed long stem alfalfa hay and corn, whereas inside BSL-3 housing calves were fed a mixture of pelleted feed, corn and alfalfa cubes. To aid in feed transition and hydration, upon movement into BSL-3 housing a combination electrolyte, probiotic powder (Bovine Bluelite^®^, TechMix, Stewart, MN) was added to the feed. Traditional cattle handling head gates were used for restraint during aerosol inoculation, blood collection, skin testing and euthanasia.

### Sample collection and lesion staging

As per the calf aerosol model [37], all calves were humanely euthanized 150 days after challenge by intravenous administration of sodium pentobarbital. Tissues were examined for gross lesions and processed for microscopic analysis and isolation of *M*. *bovis* by bacteriological culture as described [36]. The lesion severity findings and bacteriological culture results for all tissues examined are reported in detail elsewhere [36]. In brief, gross or microscopic lesions were consistently present in the tracheobronchial and caudal mediastinal lymph nodes and in one or more lung lobes in all inoculated calves. Aerosol inoculations with either isolate of *M*. *bovis* resulted in indistinguishable levels of *M*. *bovis* colonization, lesion severity, lesion distribution and immune response [36], as such all inoculated calves were considered as a single group for this analysis. Samples were fixed by immersion in 10% neutral buffered formalin and processed by standard paraffin-embedment techniques, cut in 5 μm sections and stained with hematoxylin and eosin (HE). Adjacent sections from samples containing granulomas suggestive of tuberculosis were stained by the Ziehl-Neelsen technique for visualization of acid-fast bacteria (AFB). Numerous near adjacent unstained sections were used for *in situ* cytokine expression analysis.

Not every tissue section processed for microscopic analysis contained lesions; however, one to three slides each of lymph node and lungs with tuberculoid granulomas were analyzed from each animal. For each slide, all granulomas were staged (stages I-IV) according to criteria adapted from that described previously [10–12]. These stages are defined as follows: initial (stage I), solid (stage II), necrotic (stage III) and necrotic and mineralized (stage IV).

### RNA chromogenic in situ hybridization (ISH*)*

Visualization of cytokine mRNA transcripts was done as described previously and according to manufacturer’s instructions for RNAScope^®^ 2.0 (Advanced Cell Diagnostics, Hayward, CA, USA)[38, 39] using *Bos taurus*-specific proprietary probe combinations for the following cytokines; IFN-γ (Cat #315581), TNF-α (Cat #316151), IL-10 (Cat #420941), IL-17A (Cat #406601), IL-22 (Cat #420931) and TGF-β (Cat #427271). RNAScope^®^ has been shown to be capable of single mRNA molecule detection [38]. Briefly, sections 5-μm thick, cut from formalin-fixed, paraffin embedded tissues were heated for 60 min at 60°C in a HybEZ ^™^ hybridization oven (Advanced Cell Diagnostics). Tissues were deparaffinized in xylene followed by rehydration in an ethanol series and air-dried for 5 min. Tissue sections were incubated with pretreatment 1 solution (endogenous peroxidase block) for 10 min at room temperature (RT). Slides were rinsed by immersion in double distilled water (ddH_2_O), followed by immersion in pretreatment 2 (antigen retrieval citrate buffer) for 15 min at 100–104°C (boiling). Slides were washed in ddH_2_O and pretreatment 3 (protease) applied for 30 min at 40°C. Slides were washed in ddH_2_O and target or control probes applied with incubation at 40°C for 2 hours followed by a rinse in wash buffer (Advanced Cell Diagnostics) for 2 min at RT. Signal amplification reagents 1 through 6 were serially applied for 30 min, 15 min, 30 min, 15 min, 30 min and 15 min, respectively. Slides were rinsed in wash buffer for 2 min between amplification reagents. Incubations with amplifier reagents 1 through 4 were done at 40°C, while incubations with amplifier reagents 5 and 6 were done at RT. Positive signal was visualized using Fast Red chromogenic substrate and a Gill’s hematoxylin counterstain. Slides were then dehydrated through an ethanol series to xylene. After drying 15 min at 60°C, slides were coverslipped using mounting media (EcoMount, Biocare Medical, Concord, CA, USA). The positive control probe consisted of a proprietary probe for *Bos taurus cyclophilin B* (Cat # 3194510), while the negative control probe targeted *dapB* of *Bacillus subtilis* (Cat # 312038).

### Morphometry

Slides were scanned at 40X magnification and digitized using the Aperio ScanScope XT workstation (Aperio Technology, Inc., Vista, CA, USA). Digitized images were analyzed using image analysis software (HALO^™^, Indica Labs, Inc., Corrales, NM). Each granuloma was selected as a region of interest. Using the RNA ISH HALO^™^ module the chromogenic reaction of Fast Red was identified and the signal (i.e. red dots) quantified. The RNA ISH module algorithm identifies both cells and signal allowing the quantification of signal number per area occupied by cells within the granuloma (i.e. signal / total cell area). To analyze non-granuloma areas, for each slide an area of 5 x 10^5^ μm^2^ of lung or lymph node not containing granuloma and with normal histologic appearance was analyzed. For analysis of uninfected lymph node, 5 random regions, each measuring 2 x 10^6^ μm^2^ were analyzed from 5 caudal mediastinal lymph nodes of age-matched uninfected calves.

### Statistical analysis

Mean values of signal (dots) / total granuloma area in early vs. late stage granulomas and granulomas vs. non-granuloma areas and uninfected tissues were compared using one-way analysis of variance (GraphPad Prism 6.0, GraphPad Software, San Diego, CA, USA). Tukey’s post hoc multiple comparison test was then used to compare differences between means. Mean values in lungs vs. lymph node and lung granulomas vs. non-lesion area were compared using a Student’s t-test. For all analyses *p-*value < 0.05 was considered significant.
